# Overweight, Obesity and Meningioma Risk: A Meta-Analysis

**DOI:** 10.1371/journal.pone.0090167

**Published:** 2014-02-26

**Authors:** Chuan Shao, Li-Ping Bai, Zhen-Yu Qi, Guo-Zhen Hui, Zhong Wang

**Affiliations:** 1 Department of Neurosurgery, The First Affiliated Hospital of Soochow University, Suzhou, Jiangsu, China; 2 Department of Biochemistry, Max-Planck Institute for Terrestrial Microbiology, Marburg, Hessen, Germany; Geisel School of Medicine at Dartmouth College, United States of America

## Abstract

**Background and Objectives:**

Studies of the association between excess body weight and risk of meningioma have produced inconsistent results. Therefore, a meta-analysis of published studies was performed to better assess the association between meningioma and excess body weight.

**Methods:**

A literature search was conducted in the PubMed and EMBASE databases without any limitations. The reference lists of identified articles were also screened for additional studies. The summary relative risks (RRs) and 95% confidence intervals (CI) were calculated using fixed- or random-effects models.

**Results:**

A total of 6 studies provided risk estimates for overweight or obesity. Overall, the combined RRs were 1.12 (95% CI = 0.98–1.28) for overweight and 1.45 (95% CI = 1.26–1.67) for obesity. After stratification by gender, no significant association was observed for obese men (RR = 1.30, 95% CI = 0.64–2.62), while significant association was detected for obese women (RR = 1.46, 95% CI = 1.26–1.69). No substantial differences emerged across strata of study design and geographic areas.

**Conclusion:**

The results of this meta-analysis suggest that obesity but not overweight is associated with an increased risk of meningioma. Due to the limited number of studies, further research is needed to confirm the association.

## Introduction

Meningiomas are the second most common brain neoplasms, representing approximately 20% of all intracranial tumors [1]. Most meningiomas are benign and rarely display biologically aggressive behavior [1], [2]. Despite decades of research, the aetiology of meningioma is poorly understood. Aside from certain rare genetic conditions (neurofibromatosis type I, Li Fraumeni syndrome), the only confirmed risk factor is exposure to high doses of ionizing radiation [3]–[5]. However, as the 2 types of exposures are uncommon, they can explain only a small number of the total cases. Furthermore, the incidence of meningioma has clearly risen in many Western countries [3]. Therefore, early intervention on modifiable risk factors of meningioma is very important.

Over the past several decades, obesity has emerged as a leading public health concern in the developed countries [6], [7]. Previous studies have shown that obesity contributes to increase the incidence or death of colorectal adenomas, postmenopausal breast cancer, gallbladder cancer, endometrial cancer, pancreatic cancer, renal cancer, and liver cancer [8], [9]. However, the relationship between meningioma and obesity is still unclear. In recent years, a number of studies have explored the association between the risk of meningioma and excess body weight, but the results were conflicting [10]–[21]. Several studies indicated that excess body weight was associated with a higher risk of meningioma [10]–[12], [14], [16], whereas no significant association was reported in other studies [13], [15], [17]–[21]. This discrepancy in the results may result from different characteristics of subjects or study methodologies. Moreover, no quantitative summary of the evidence has ever been reported. Therefore, a meta-analysis of published cohort and case-control studies was conducted to quantify the effect of obesity and overweight on the occurrence of meningioma.

## Materials and Methods

### Search Strategy

Two reviewers (CS and ZYQ) independently performed a literature search of the PubMed and EMBASE databases without any limitations on language and publication date. The following search terms were used: “body mass index”, “overweight”, “obesity”, “body weight”, “body size”, “anthropometry”, and “adiposity” combined with “meningioma”, “brain cancer”, “brain tumor”, and “brain neoplasm”. We also reviewed the reference lists of included articles for additional studies. The last updated search was performed on August 23, 2013.

### Study Selection

Studies were identified for this meta-analysis if they fulfilled all the following inclusion criteria: (1) used a case-control or cohort design; (2) clear description of overweight or obesity defined by body mass index (BMI) in kg/m^2^; (3) assessed the relationship between risk of meningioma and overweight or obesity; (4) reported estimates of relative risk [odds ratio (OR), hazard ratio (HR)] with corresponding 95% CIs or sufficient data to estimate them; (5) in the case of multiple reports of the same study population, only the most recent and informative one was included; (6) we excluded those studies in which non-obese people were reference subjects because non-obese people include a number of overweight people; and (7) we also excluded those studies that involved total brain tumors because brain tumors are a heterogeneous group of tumors that vary in tissue origins, invasive potential and prognosis.

### Data Extraction

Two authors (CS and ZYQ) independently abstracted the following data in a standard format: the first author, publication year, country in which performed, study period, age range of participants, sex, number of subjects (cases, controls or cohort size), meningioma diagnosis method, measure of exposure, risk estimates and corresponding 95% CI, and matching and adjustments. Any disagreements were resolved by discussion.

### Assessment of Methodological Quality

The Newcastle-Ottawa Scale (NOS) for assessing the quality of observational studies was used to assess the quality of included studies [22]. The NOS is based on three major components: selection of the study groups (0–4 stars), comparability of cases and controls (0–2 stars), or cohorts, and ascertainment of exposure/outcome (0–3 stars). A study awarded 6 stars or more is considered a high-quality study.

### Statistical Analysis

The RR was used as the measure of the relationship between meningioma and overweight or obesity. Because meningioma is rare, ORs and HRs were accurate approximations of RRs [23]. In this meta-analysis, the most fully adjusted risk estimates were used; however, if such estimates were unavailable, crude effect estimates with 95% CIs were included. In this meta-analysis, we only reported the risk estimates based on the baseline data. Heterogeneity among studies was assessed by Cochran’s Q and I^2^ statistics [24], [25]. For Cochran’s Q statistic, substantial heterogeneity was defined as P<0.1 [24]. The I^2^ statistic ranges in value from 0 to 100% (I^2^<25%, low heterogeneity; I^2^ = 25%–50%, moderate heterogeneity; and I^2^>50%, high heterogeneity) [25]. Both the fixed- and random-effects models were used to calculate the pooled RR [26]. If substantial heterogeneity was found, we presented the results from random-effects models. Subgroup analyses were conducted according to study design (case-control and cohort), gender (male and female), and geographic regions (Europe and North America). A sensitivity analysis was performed to assess the influence of the individual studies on the overall results by omitting one study at a time. Publication bias was assessed by Egger’s test (P<0.05 was considered significant) [27].

We defined body mass categories according to the World Health Organization (WHO) guidelines: underweight (BMI<18.5 kg/m^2^), normal weight (BMI≥18.5 and <25 kg/m^2^), overweight (BMI≥25 and <30 kg/m^2^), and obesity (BMI≥30 kg/m^2^). In this meta-analysis, normal weight was used as the reference category. When non-standard categories of BMI were reported, we selected the category that most closely approximated those defined by the WHO guidelines. When more than one estimate in a study fell into the range representing overweight or obesity, we calculated a combined risk estimate using the method proposed by Hamling et al [28]. All statistical analyses were performed using STATA, version 11.0 (STATA, College Station, TX, USA).

## Results

### Literature Search and Study Characteristics

Fig. S1 shows a flow diagram for the selection process. A total of 2607 potentially relevant studies were identified from the initial search. After a careful review, the remaining 26 articles were considered of interest and their full-text was assessed for eligibility. Of 26 studies, 20 were excluded after reading the full-text [10], [14], [15], [17], [18], [20], [29]–[42]. The major reasons for excluding these studies were as follows: evaluating overweight and obesity together (n = 2) [15], [18], no available data [20], obesity measured by Quetelet index, Cohen’s Kappa index or weight (n = 2) [10], [17], non-obese people as the reference (n = 1) [14], and involving total brain tumor in their subjects (n = 14) [29]–[42]. Thus, a final total of 6 studies (4 cohort studies and 2 case-control studies) were included in this meta-analysis [11]–[13], [16], [19], [21]. The range of publication periods for the included studies was 2006–2013. All studies were published in English. Of 6 studies, 3 were performed in North America [12], [13], [16] and 3 in Europe [11], [19], [21]. Two studies included women and men [19], [21] and 4 studies included women only as subjects [11]–[13], [16]. The data on weight and height were collected through self-reporting [11]–[13], [16], measurement [21], or both of the 2 methods [19]. The definition of cases was based on the radiological criteria or pathology reports. Additional characteristics of the included studies are shown in Table 1. The quality of the included studies was evaluated by NOS. Table S1 shows the results of the assessment of methodological quality. All included studies obtained more than six stars, suggesting that the overall quality of the studies is good.

**Table 1:** Characteristic of the included studies in this meta-analysis.

First author, Publication year	Country^a^	Study period	Age	Sex	Cases/Cohort	Case diagnosis	measurement method	Matching or adjustment
**Cohort studies**								
Benson, 2008	5	1996–2001/6.2	50–65	F	390/1,249,670	Cancer registry	Self-reported	Age, height, strenuous exercise, socioeconomic level, smoking, alcohol intake, parity, age at first birth, OC
Johnson,2011	1	1986–2004/10.5	55–85.7	F	125/291,021	Medicare data	Self-reported	Age.
Michaud, 2011	2–11	1991–2004/8.4	35–70	M/F	203/380,775	Cancer registry	Self-reported, Measured	Age, country, sex, education.
Wiedmann,2013	4	1984–1986/23.5	≥20	M/F	81/74,242	Cancer registry	Measured	Age, sex
**Case-control studies**								
Claus,2013	1	2006–2011	29–79	F	1,127/1,092	Cancer registry	Self-reported	Age, sex, residence, race, education, menopause status, age at menopause, age at menarche, smoking, alcohol use, breastfeeding, OC, HRT, number of FLB, age at FTP.
Custer,2006	1	1995–1998	≥18	F	143/286	Pathology reports	Self-reported	Age, race, marital status

M, male; F, female; OC, oral contraceptive; HRT, hormone replacement therapy; FLB, first live birth; FTP, full-term pregnancy.

a Studies were conducted in: (1) USA, (2) Sweden, (3) Denmark, (4) Norway, (5) United Kingdom, (6) France, (7) Netherlands, (8) Spain, (9) Italy, (10) Germany, (11) Greece.

### Meta-analysis Results

**Figure 1:**
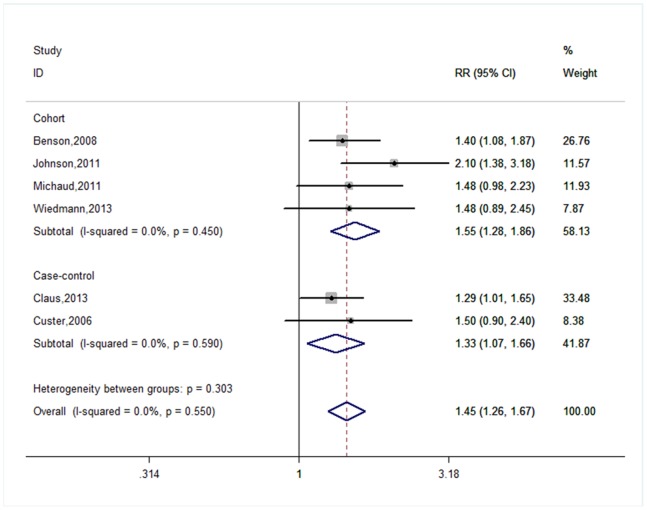
Forest plot for obesity versus normal weight.

Figure 1 shows the forest plots for obesity versus normal weight. The summary RRs for case-control, cohort studies, and all studies were 1.33 (95% = 1.07–1.66, P_Heterogeneity_ = 0.590, I^2^ = 0.0%), 1.55 (95% = 1.28–1.86, P_Heterogeneity_ = 0.450, I^2^ = 0.0%), and 1.45 (95% CI = 1.26–1.67, P_Heterogeneity_ = 0.550, I^2^ = 0.0%), respectively. In subgroup analyses by gender, a statistically significant link between the risk of meningioma and obesity was observed for females (RR = 1.46, 95% CI = 1.26–1.69, P_Heterogeneity_ = 0.515, I^2^ = 0.0%), but not for males (RR = 1.30, 95% CI = 0.64–2.62, P_Heterogeneity_ = 0.427, I^2^ = 0.0%). In subgroup analyses by geographic regions, the pooled results were significant in both North American studies (RR = 1.47, 95% CI = 1.21–1.78, P_Heterogeneity_ = 0.142, I^2^ = 48.7%) and European studies (RR = 1.43, 95% CI = 1.16–1.77, P_Heterogeneity_ = 0.967, I^2^ = 0.0%).

**Figure 2:**
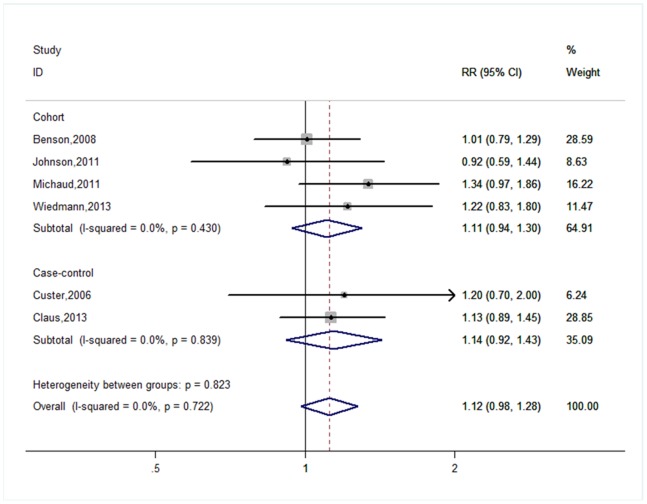
Forest plot for overweight versus normal weight.

Figure 2 shows the forest plots for overweight versus normal weight. The pooled results based on all studies suggested there was no significant association between risk of brain tumor and overweight (RR = 1.12, 95% CI = 0.98–1.28, P_Heterogeneity_ = 0.722, I^2^ = 0.0%). In subgroup analyses, we found that the associations between overweight and risk of meningioma were not significantly modified by gender, geographic regions, or study design (Table 2).

**Table 2:** Summary risk estimates of the association between BMI and meningioma risk.

Group	Overweight (25≤BMI≤29.9 kg/m^2^)				Obesity (BMI≥30 kg/m^2^)			
			Heterogeneity				Heterogeneity	
	Number ofstudies	RR(95% CI)	I^2^	P	Number ofstudies	RR(95% CI)	I^2^	P
All studies	6	1.12(0.98–1.28)	0.0%	0.722	6	1.45(1.26–1.67)	0.0%	0.550
**Study design**								
Case-control	2	1.14(0.92–1.43)	0.0%	0.839	2	1.33(1.07–1.66)	0.0%	0.590
Cohort	4	1.11(0.94–1.30)	0.0%	0.430	4	1.55(1.28–1.86)	0.0%	0.450
**Gender**								
Male	2	1.03(0.64–1.66)	0.0%	0.603	2	1.30(0.64–2.62)	0.0%	0.427
Female	6	1.13(0.98–1.29)	0.0%	0.573	6	1.46(1.26–1.69)	0.0%	0.515
**Geographic area**								
Europe	3	1.14(0.96–1.36)	0.1%	0.368	3	1.43(1.16–1.77)	0.0%	0.967
North America	3	1.09(0.90–1.33)	0.0%	0.682	3	1.47(1.21–1.78)	48.7%	0.142

### Sensitivity Analysis

**Figure 3:**
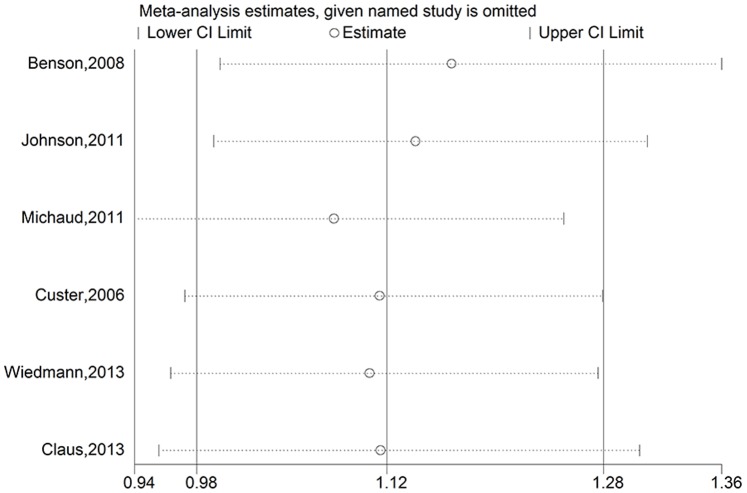
Sensitivity analyses for overweight versus normal weight.

**Figure 4:**
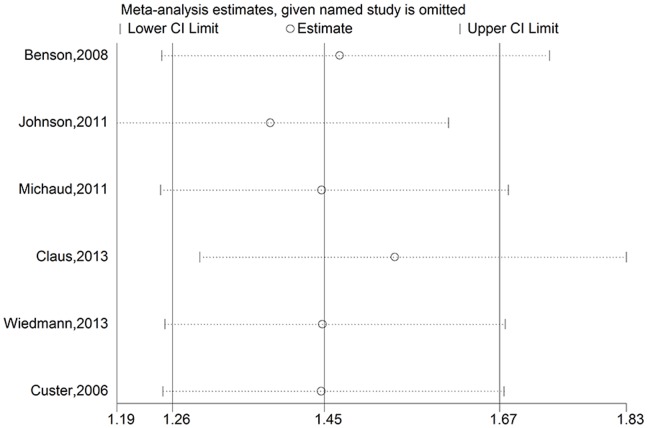
Sensitivity analyses for obesity versus normal weight.

To assess the stability of the results of the meta-analysis, sensitivity analyses were conducted by excluding one study at a time. For overweight, a borderline significant association was found after omitting the Million Women Study [11] and the Iowa Women’s Health Study [16]. The pooled RRs were 1.17 (95% CI = 1.00–1.36, P_Heterogeneity_ = 0.752, I^2^ = 0.0%) for excluding the Million Women Study and 1.14 (95% CI = 0.99–1.31, P_Heterogeneity_ = 0.728, I^2^ = 0.0%) for excluding the Iowa Women’s Health Study (Figure 3). The other results of sensitivity analyses for overweight were not significantly altered (data not shown). For obesity, none of the results was significantly altered, indicating that our results were robust (Figure 4).

### Publication Bias

The results of Egger’s test suggest that no evidence of publication bias was observed (P = 0.204 for obesity and P = 0.764 for overweight).

## Discussion

This meta-analysis of 4 cohort studies and 2 case-control studies assessed the association of meningioma with obesity or overweight. Our analysis identified an association between an increased risk of meningioma and obesity. However, no significant correlation with overweight was observed. In further analyses by gender and geographic area, similar trends were observed.

Several potential mechanisms have been proposed to explain how obesity can contribute to the development of meningioma, although the exact biological mechanisms are unclear. Currently, the most well-known mechanism is the insulin-like growth factor (IGF) hypothesis of obesity-related cancer [8], [43]–[45]. Obesity is associated with insulin resistance and hyperinsulinemia, which reduce the levels of insulin-like growth factor binding protein 1 (IGFBP-1) and insulin-like growth factor binding protein 2 (IGFBP-2). The decrease in these proteins leads to higher circulating concentrations of free or bioactive insulin-like growth factor 1 (IGF-1) and a change in cell environment that stimulates tumor growth and inhibits apoptosis. Furthermore, the involvement of the IGF system in brain development has been demonstrated by in vitro and in vivo studies [46], [47]. Finally, laboratory studies have confirmed that IGF1, IGF2, and IGF1R genes are overexpressed in meningioma [46]. Other possible mechanisms include chronic inflammation, alterations in adipokine concentrations and sex hormones, sharing genetic susceptibility, obesity-related hypoxia, and migrating adipose stromal cells [44], [48].

In this meta-analysis, we further investigated the correlation with obesity separately for females and males. The results of subgroup analyses show that obesity was associated with a significantly elevated risk of meningioma in females, but not in males. The potential explanations for the sex difference might be related to the effect of sex hormones. Obesity is positively associated with circulating concentrations of testosterone in females [49], [50], but inversely associated with testosterone concentrations in males [51], [52]. There is evidence that testosterone promotes cell proliferation and local production of IGF-I and IGF-I-R [53]. Moreover, estrogens also interact with IGF, which stimulates tumor growth and prohibits cell apoptosis [44]. Recently, a meta-analysis of 11 studies has suggested that the use of hormone replacement therapy is correlated with an increased risk of meningioma in women [54]. In our meta-analysis, many subjects have implied that they used female hormone when their menstrual cycle ended [12], [13], [16]. Thus, it is conceivable that obese females bear a larger risk of meningioma than obsess males. An alternative explanation for observed gender differences is that these findings may have occurred by chance because a limited number of studies were involved in subgroup analyses. Therefore, further evaluation of obesity relative to risk of meningioma is needed with more attention to the influence of gender.

Two cohort studies have examined the association between waist-hip ratio (WHR) and risk of meningioma: the Iowa Women’s Health Study (IWHS) [16] and the European Prospective Investigation into Cancer and Nutrition (EPIC) [19]. Michaud and colleagues in the EPIC found that abdominal obesity (defined as WHR) was associated with an increased risk of meningioma, although these correlations were not statistically significant [19]. In the latter study, a similar trend was detected for meningioma [16]. Compared with BMI, WHR is considered to be a more accurate index of obesity because the WHR takes the anatomic distribution of body fat in account and distinguishes lean muscle mass from fat mass [55]–[57]. Therefore, both BMI and WHR should be considered in future studies.

When obesity was found to be closely related to a higher risk of meningioma, several researchers proposed the hypothesis that underweight is related to a low risk of meningioma. To our knowledge, only 3 studies to date have analyzed the relationship between the risk of meningioma and underweight [18], [19], [21]. A hospital-based case-control study with 479 participants found no significant positive association (OR = 1.3, 95% CI = 0.6–3.0) between meningioma and underweight (defined by BMI<19 kg/m^2^) [18]. However, an inverse result was observed in the Nord-Trøndelag Health Study [21]. This prospective study showed that underweight (defined by BMI<20 kg/m^2^) was not meaningfully correlated with a lower risk of meningioma (RR = 0.67, 95% CI = 0.29–1.56) [21]. In EPIC, no significant association was detected (RR = 1.00, 95% CI = 0.46–2.19) [19]. These findings may be chance results due to the limited number of subjects, various study designs, and non-standard definitions of underweight used. Hence, additional well-designed studies are warranted to better understand the association between underweight and the risk of meningioma.

Several potential limitations of this meta-analysis should be noted. First, our meta-analysis was based on the small number of studies. Indeed, a great number of studies have evaluated the relationship between obesity and the risk of brain tumors [29]–[42]. However, brain tumors are a heterogeneous group of tumors that vary in tissue origins, invasive potential and prognosis. Thus, these studies cannot be included in this meta-analysis and further evaluation of obesity with risk of brain tumors is needed with particular attention to stratification by the type of tumor. Second, as all included studies were observational, we cannot exclude the possibility that our findings could be due to unmeasured or residual variables. Third, the estimation of weight and height in most of included studies was based on subjects’ self-reporting. It is possible that the weight has been underreported, particularly by overweight or obese individuals, and that height has been overestimated. Thus, this factor might have resulted in a degree of underestimation of the true associations. Fourth, because no studies involved Chinese/Asian populations, additional investigations in non-Western countries are warranted to extend the current findings [58]. Fifth, obesity may not be the main causative factor because obesity could be a consequence of other causative factors, for example, sex hormones and unhealthy lifestyles (i.e., smoking, heavy alcohol consumption and less exercise). The involvement of female hormones in meningioma carcinogenesis has been demonstrated in experimental and histopathologic studies as well as observational studies [59]–[64]. Additionally, the unhealthy lifestyles listed above have generally been considered to increase the risk of cancer. Finally, publication bias is often a concern in a meta-analysis because null results tend to be unpublished.

In summary, the results of this meta-analysis show that obesity is positively associated with the risk of meningioma. These findings also indicate that maintaining a healthy body weight may, in part, prevent the occurrence of meningioma.

## Supporting Information

Figure S1 **Flow diagram of study selection.**
(TIF)

Table S1 **Methodological quality of included studies based on the Newcastle–Ottawa Scale.**
(DOCX)

Checklist S1 (DOC)
