# Supplementary figures and images for: Overweight, Obesity and Meningioma Risk: A Meta-Analysis
Source: PLoS One. 2014 Feb 26;9(2):e90167. doi: 10.1371/journal.pone.0090167 (PMC3935973; doi:10.1371/journal.pone.0090167)

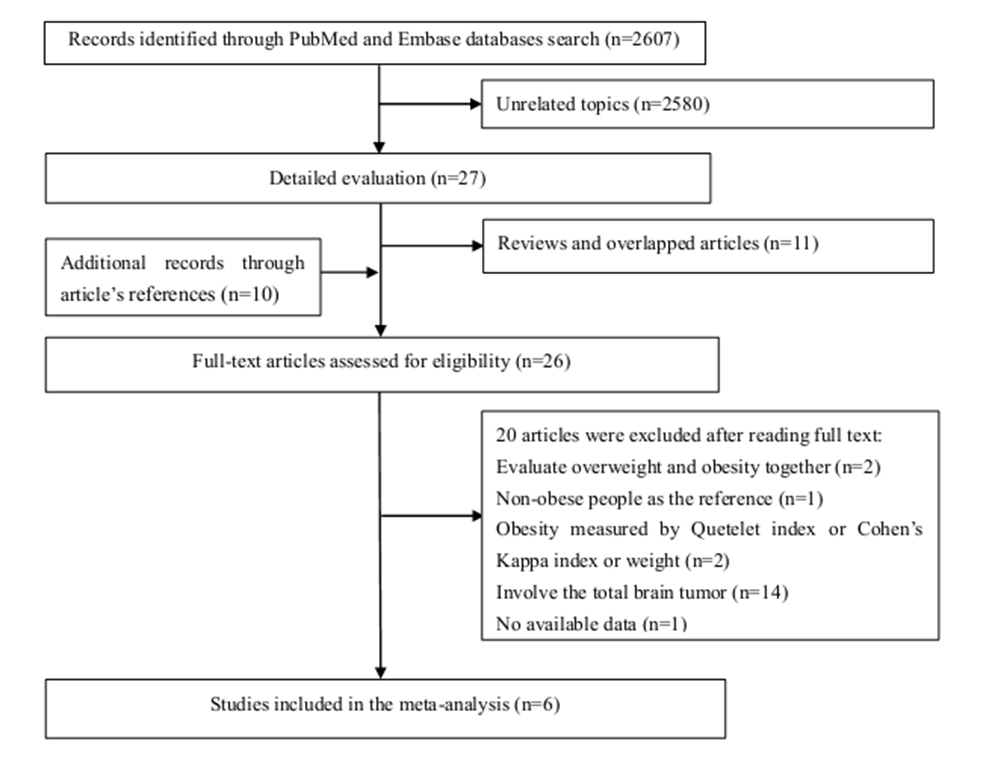

Supplement: Figure S1 — Flow diagram of study selection. (TIF) [file pone.0090167.s001.tif]
