# Supplementary material for: Overweight, Obesity and Meningioma Risk: A Meta-Analysis
Source: PLoS One. 2014 Feb 26;9(2):e90167. doi: 10.1371/journal.pone.0090167 (PMC3935973; doi:10.1371/journal.pone.0090167)
Supplement: Table S1 — Methodological quality of included studies based on the Newcastle–Ottawa Scale. (DOCX) [file pone.0090167.s002.docx]

Table S1 Methodological quality of included studies based on the Newcastle–Ottawa Scale

| First author, Publication year | Selection | Comparability | Outcome/  Exposure | Total |
| --- | --- | --- | --- | --- |
| Cohort studies |  |  |  |  |
| Benson,2008 | *** | ** | *** | 8* |
| Johnson,2011 | *** | * | *** | 7* |
| Michaud,2011 | *** | ** | *** | 8* |
| Wiedmann,2013 | **** | ** | *** | 9* |
| Case-control studies |  |  |  |  |
| Custer,2006 | **** | ** | ** | 8* |
| Claus,2013 | **** | ** | ** | 8* |

Table S2
